# Torque teno virus viral load is related to age, CMV infection and HLA type but not to Alzheimer's disease

**DOI:** 10.1371/journal.pone.0227670

**Published:** 2020-01-09

**Authors:** Gabriel Westman, Catherine Schoofs, Martin Ingelsson, Josef D. Järhult, Shaman Muradrasoli

**Affiliations:** 1 Department of Medical Sciences, Section of Infectious Diseases, Uppsala University, Uppsala, Sweden; 2 Department of Laboratory Medicine, Karolinska Institutet, Stockholm, Sweden; 3 Department of Public Health and Caring Sciences, Uppsala University, Uppsala, Sweden; Cincinnati Children's Hospital Medical Center, UNITED STATES

## Abstract

Torque teno virus (TTV) is an unenveloped, circular, single stranded DNA virus with a genome size of approximately 3.8 kb. Previous studies have demonstrated varying grades of association between TTV DNA levels and immune deficiencies related to age, chronic infections and cancer. Alzheimer's disease (AD) has been related to persistent viral infections such as HSV-1 and CMV, but it is not known whether TTV viral load could serve as a functional biomarker of cellular immunity in this setting. Therefore, the objective of this study was to investigate whether TTV infection and viral load is related to AD status, CMV immunity, systemic inflammation or HLA types connected to anti-viral immunity. A total of 50 AD subjects and 51 non-demented controls were included in the study. AD subjects were diagnosed according to NINCDS-ADRDA and DSM-IV criteria and neuroradiologic findings were consistent with the diagnosis. TTV viral load was analyzed in plasma samples using a quantitative real-time PCR. Using a cut-off for TTV status at 200 copies/ml, 88% (89/101) of the study subjects were classified as TTV positive. TTV viral load significantly increased with age (beta 0.049 per year, p<0.001) but significantly decreased in relation to CMV IgG levels (beta -0.022 per 1000 units, p = 0.005) and HLA-B27 positivity (beta -0.53, p = 0.023). In conclusion, TTV immune control is not significantly affected by AD status, but appears related to age, CMV humoral immune response and HLA type.

## Introduction

Torque teno virus (TTV) is an unenveloped, circular and single stranded DNA virus with a genome size of approximately 3.8 kb [1]. The virus, belonging to the *Anelloviridae* family, was first identified in 1997 in the serum of a Japanese patient who suffered from post-transfusion hepatitis [2]. A total of 29 TTV genotypes have been determined and classified in the genus *Alphatorquevirus* [3]. TTV DNA is detectable in 70 to 90 percent of the human population and viral load is usually in the range of 3–6 log10 copies/ml [4]. TTV infection has not been shown to cause clinical disease, but the virus has been associated with many viral co-infections such as persistent human cytomegalovirus (CMV), human immunodeficiency virus (HIV) and hepatitis C virus (HCV) [4, 5].

Previous studies have demonstrated an association between TTV viral load and deficiencies of the immune system due to chronic infections and cancer [6–9]. Also, a significant increase of TTV DNA levels has been shown to occur after iatrogenic immunosuppression and TTV DNA load has therefore been suggested as a surrogate marker of the immunological status of the host [10]. Recently, in an Italian cohort of 379 elderly subjects, TTV DNA levels were found to correlate with age. Also, the study proposed TTV DNA viral load ≥4.0 log10 in polymorphonuclear leukocytes as a predictive factor for all-cause mortality among elderly [11]. However, other studies have shown significantly higher TTV viral load both in very young and elderly subjects [12]. Taken together, these findings suggest that TTV viral load could potentially serve as a functional biomarker of general anti-viral immune capacity.

There is an increasing amount of evidence that Alzheimer's disease (AD), in its most common sporadic late-onset form, is an immunological disease with a possible causal relation to latent or chronically persistent CNS infections. Amyloid beta, forming the plaques that are one of the neuropathological disease hallmarks, have now been characterized as an antimicrobial peptide [13]. Beside ApoE4, the strongest genetic predictor, epidemiologic associations have been made both to herpes simplex virus type 1 (HSV-1) and CMV indicating that latent or chronically persistent infections could be correlated or even causative in the AD pathophysiological process [14, 15]. While HSV seems to infect brain tissue and directly promote amyloid beta production, CMV is thought to promote AD pathology through its immune modulation where the T-lymphocyte phenotype is shifted towards a more effector-oriented cellular immune response [16, 17]. It is hitherto not known how this interplay between persistent viral infections, immunity and AD is reflected in terms of TTV immune control. Given that TTV viral load has been previously proposed as a functional biomarker of anti-viral immunity, TTV immune control could serve as a prognostic factor in early phases of disease and provide further insight in the AD pathophysiological process. The objective of this study was to investigate whether TTV infection and viral load are related to AD status, CMV infection, biomarkers associated with systemic inflammation and HLA types related to anti-viral immunity [18–21].

## Materials and methods

### Subjects, sampling and routine analyses

A total of 50 AD patients and 52 non-demented (ND) controls from a previously described cohort were included in the study [17]. One sample from the ND group was lost prior to analysis, rendering a total of 50 AD study subjects and 51 ND controls. All AD subjects had prior to inclusion been diagnosed with AD according to NINCDS-ADRDA and DSM-IV criteria and neuroradiologic findings were consistent with the diagnosis. Subjects in the ND control group were recruited via local advertising and did not show any clinical signs of cognitive deficits or complaints of subjective cognitive impairment. Blood samples were acquired by venipuncture and plasma was separated from blood cells through centrifugation. Samples were temporarily stored at -20°C, whereafter they were long-term stored at -70°C until analysis. The participants’ dementia status was blinded during the laboratory work.

Clinical chemistry analyses including CRP, IL-6 and Apolipoprotein E (*APOE*) genotyping, were performed at the Department of Clinical Chemistry and Pharmacology, whereas HLA genotyping (PCR-SSO) was performed at the Department of Clinical Immunology and Transfusion Medicine, both accredited laboratories at Uppsala University Hospital. CMV IgG was analyzed using SIEMENS Enzygnost anti-CMV/IgG, at the Department of Clinical Microbiology at Uppsala University Hospital, and study participants were classified as either CMV seropositive or seronegative.

This study was carried out in accordance with the Declaration of Helsinki. Written informed consent was obtained from all study participants, together with consent from a close relative if the subject was considered incapable of taking his or her own decision. The study was approved (no. 2009/097) by the Regional Ethical Review Board in Uppsala, Sweden.

### TTV DNA quantification

Total nucleic acid was extracted from 200 μl of plasma of 101 patient samples using MagNa Pure 96 (Roche) and was stored at -20°C until use. TBE-buffer (Tris-borate-EDTA) was included in each extraction as process control. A previously published TaqMan realtime PCR targeting a highly-conserved segment of the untranslated region (UTR) of the viral genome was used to quantify TTV DNA load [9, 22]. The following primers and probe were used for PCR amplification: forward primer 5′-GTGCCGIAGGTGAGTTTA-3′, position 177–194; reverse primer 5′-AGCCCGGCCAGTCC-3′, position 226–239; probe 5′FAM-TCAAGGGGCAATTCGGGCT- TAMRA3′, position 205–223. TTV DNA quantifications were performed using a TaqManTM Fast Universal PCR Master Mix, no AmpErase^™^ UNG on an ABI 7500 instrument (Applied Biosystems) as previously described by Maggi and adapted by Görzer et al [9, 22]. The PCR reaction was carried out slightly modified according to following conditions; total volume of 25 μl containing 5 μl DNA 400 nM of each primer and 20 nM of the probe. The PCR profile consisted of 1 cycle at 95°C for 10 minutes followed by 45 cycles at 95°C for 15 seconds and 58°C for 60 seconds.

Plasmid DNA with cloned TTV fragment was used as a standard to determine the sensitivity and dynamic range of the assay. Amplification of 10-fold serial dilutions of the plasmid standard ranging from 200 to 10^6^ copies per reaction was carried out. A linear correlation between Ct values and the log10 of the initial concentration of the plasmid DNA quantities was observed in the range 10^2^–10^6^ copies per reaction. Serial dilutions of plasmids containing 2 × 10^6^ copies/μl to 2 × 10^2^ copies/μl were included in each analysis, as well nuclease-free H_2_O, the process control as a negative controls. Sensitivity tests was performed by titration of plasmid DNA and titration of a positive sample in triplicates. The lower detection limit was 200 copies/μl, and the linear range of the assay was 10^2^ to 10^6^ copies/μl and the R^2^-value was 0.9997. Considering the dilution factor of the extraction and amplification a conversion factor of 2500 was applied to compensate for dilutions, in order to provide the final viral load results in copies/ml, taking in account all dilutions from extraction to quantification including the100-fold dilution of the extracted DNA due to inhibition.

The specificity of the assay was tested by analyzing generated PCR products on gel electrophoresis and bands corresponding to the expected sizes of the target were seen, and no nonspecific amplification products were present. Furthermore, 8 PCR products was sequenced, and BLAST search showed that all 8 to be TTV.

### Statistical analyses

R version 3.4.1 (The R Foundation for Statistical Computing) with packages plyr (version 1.8.4) and ggplot2 (version 3.0) was used for statistical analysis and graphical output.

After visual inspection of the data distribution, but prior to statistical modelling, TTV viral load data was log10-transformed. In addition to AD status, predictor variables were selected based on previous findings by Haloschan et al. [4], along with a biological rationale that TTV infection in an Alzheimer's disease setting could be related to biomarkers of systemic inflammation, HLA alleles previously connected to antiviral immune response and ApoE genotype. Prior to any statistical analyses, HLA-B27 and HLA-B57 status were selected from the HLA-typing dataset for inclusion in the regression models. Initially ApoE alleles E2/E3/E4 were included in the analysis, but due to the allele frequency in the cohort ApoE analysis was limited to ApoE4 status.

Predictors of TTV status was analysed using univariable logistic regression, while predictors of TTV viral load were analysed using a multivariable linear regression model including all remaining parameters except for MMSE. P-values below 0.05 were considered significant.

## Results

### Demographics, clinical characteristics and viral load

An overview of the cohort demographics, clinical characteristics and TTV DNA levels is presented in Table 1.

**Table 1:** Demographics, clinical characteristics and TTV viral load.

Continuous data reported as median (IQR)	AD (N = 50)	ND (N = 51)
Age (years)	78 (73–83)	75 (72–80)
Sex (male:female)	28:22	22:29
Mini-mental State Examination score	20 (17–23)	NA
CMV IgG positive	42/50	40/51
CMV IgG	17000 (8950–26750)	13000 (2350–25000)
HLA-B27 positive	6/50	10/51
HLA-B57 positive	2/50	3/51
ApoE4 positive	32/50	18/51
CRP (mg/L)	1.1 (0.59–2.7)	1.3 (0.59–3.35)
IL-6 (ng/L)	1.05 (0.5–2.5)	1.2 (0.6–3.05)
TTV DNA (log10 copies/ml)	5.99 (5.41–6.38)	5.85 (4.57–6.22)

### TTV viraemia vs. AD status

**Fig 1:**
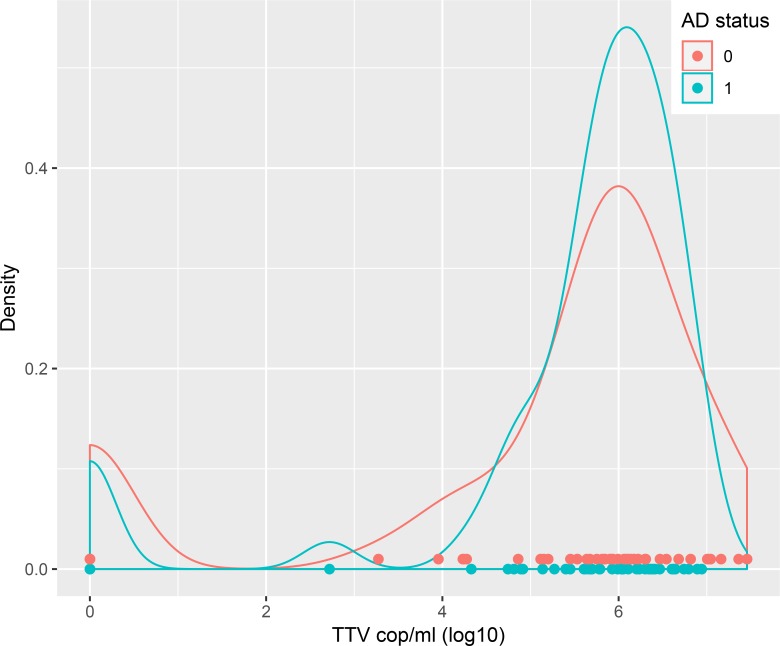
Density plot of TTV viral load. The distribution of viral load indicates good separation between TTV positive and negative populations. Alzheimer status coded in blue (AD) and red (ND). TTV DNA levels were offset by one unit to allow log transformation of zero values.

TTV DNA levels were compared between AD and ND subjects using density plots, showing no obvious differences between groups. The density plots indicated a separation between TTV positive and negative subjects in line with the assay sensitivity at 200 copies/ml and hence cut-off for TTV status was set accordingly. Using this cut-off, 88% (89/101) of subjects were classified as TTV positive (Fig 1).

### Predictors of TTV status

Clinical characteristics, CMV serostatus, CMV IgG, HLA type (B27 and B57), ApoE4 genotype and biomarkers of systemic inflammation (CRP, IL-6) were analysed in relation to TTV status using univariable logistic regression, showing no significant correlation (Table 2).

**Table 2:** Univariable logistic regression analysis of TTV status predictors. All subjects included in analysis.

	Odds ratio	Lower CI (2.5%)	Upper CI (97.50%)	p-value
**AD status**	2.14	0.60	7.62	0.24
**Sex (male)**	1.43	0.42	4.85	0.56
**Age**	1.06	0.99	1.14	0.12
**CMV serostatus**	2.47	0.66	9.25	0.18
**CMV IgG (1000 units)**	1.00	0.96	1.04	0.82
**HLA-B27**	0.51	0.12	2.15	0.36
**HLA-B57**	0.52	0.05	5.06	0.57
**ApoE4**	1.43	0.42	4.85	0.56
**CRP**	1.13	0.88	1.44	0.35
**IL-6**	1.32	0.83	2.09	0.24

### Predictors of TTV viral load in TTV positive subjects

**Fig 2:**
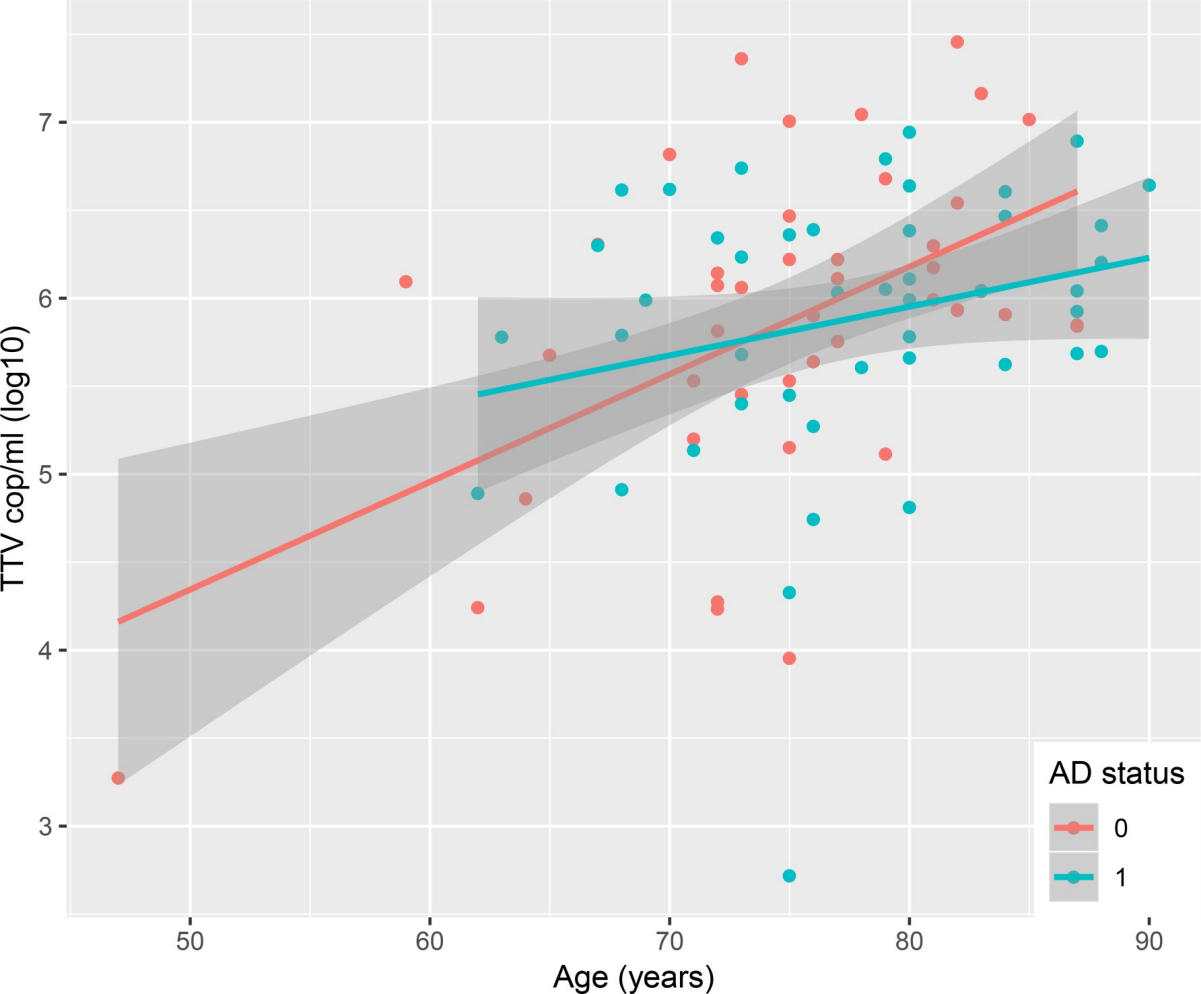
TTV viral load vs. age. TTV viral load increases with age. Alzheimer status coded in blue (AD) and red (ND).

**Fig 3:**
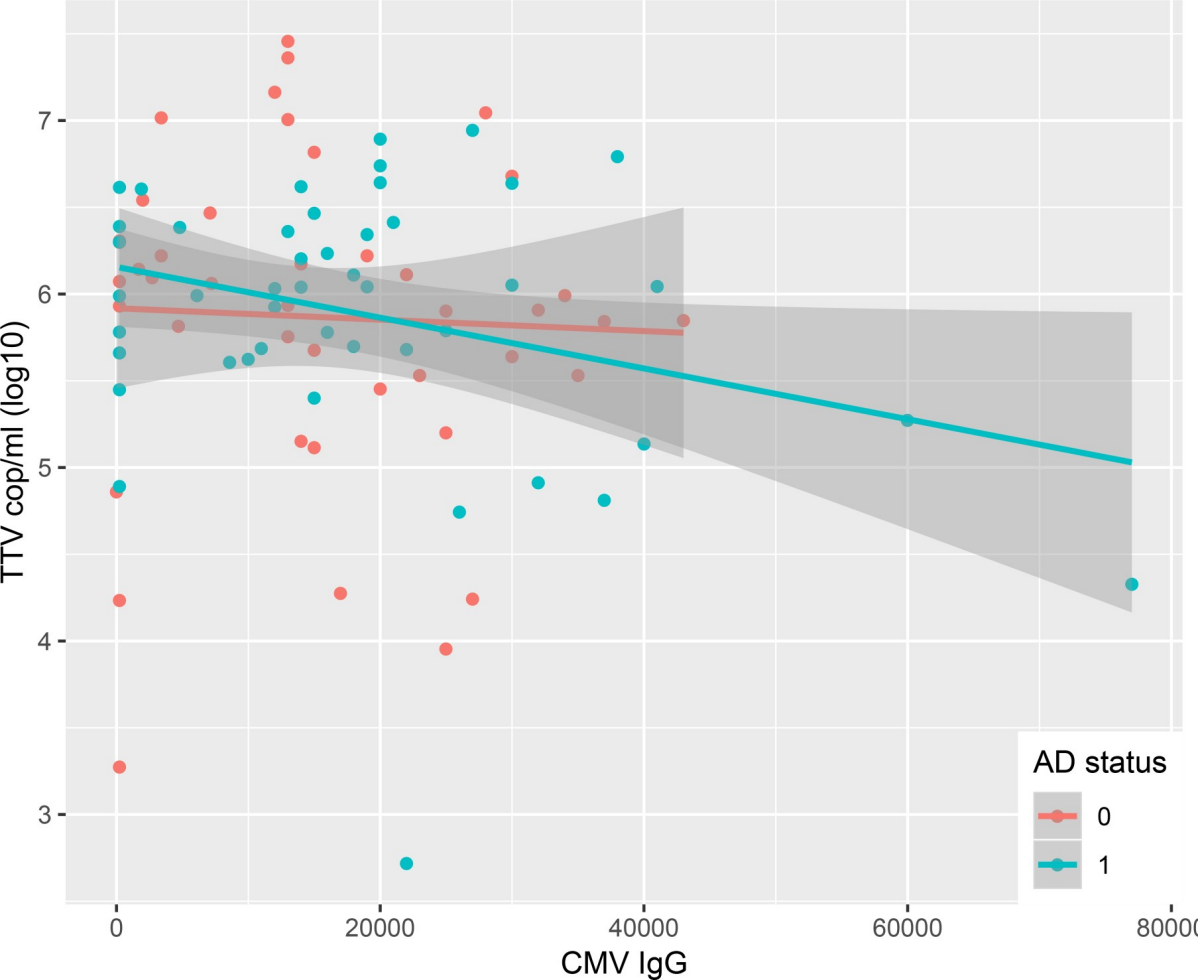
TTV viral load vs. CMV IgG. TTV viral load decreases with increasing CMV IgG. Alzheimer status coded in blue (AD) and red (ND).

In a multivariable linear regression model including only TTV positive subjects, the same predictor variables as described above were analysed for correlation to log10-transformed TTV DNA levels (Table 3). Age (p<0.001, Fig 2) correlated to increasing TTV viral load whereas CMV IgG (p = 0.005, Fig 3) and HLA-B27 (p = 0.023) correlated to lower TTV DNA levels.

**Table 3:** Multivariable linear regression model of TTV viral load predictors. Only TTV positive subjects included in the analysis.

	Beta estimate	Lower CI(2.5%)	Upper CI (97.5%)	p-value
**Dementia**	-0.094	-0.43	0.24	0.58
**Sex (male)**	0.046	-0.30	0.40	0.79
**Age**	0.049	0.025	0.072	<0.001
**CMV serostatus**	0.41	-0.14	0.97	0.14
**CMV IgG (1000 units)**	-0.022	-0.037	-0.0066	0.005
**HLA-B27**	-0.54	-1.01	-0.076	0.023
**HLA-B57**	0.53	-0.25	1.32	0.18
**ApoE4**	0.11	-0.24	0.45	0.54
**CRP**	-0.0016	-0.031	0.027	0.92
**IL-6**	0.011	-0.046	0.068	0.71

## Discussion

Our finding of 88% of subjects being TTV DNA positive is in line with previous studies, describing a TTV DNAemia prevalence in the general population ranging between 68 and 95% [23–25]. In the absence of a serologic gold standard for TTV carrier status, it remains an open question whether non-viraemic subjects are uninfected or whether there could be elite controllers of TTV among these. In most studies where TTV DNA have been quantified by real-time PCR, the viral load was in the range of 1×10^2^ copies/ml to 1×10^6^ copies/ml, which is also in agreement with our results. However, even higher viral loads have previously been described in cases of co-infection of TTV and other viruses such as HIV, HCV and EBV as well as in the elderly [4, 26, 27].

TTV DNA has been proposed as a functional biomarker of immunity in a post-transplant setting [9], whereas less is known about TTV DNA levels in other clinical settings. In this study TTV viral load increased with age but decreased with CMV IgG levels and HLA-B27 status. This is partly in contrast to previous findings by Haloschan et al [4]. We could indeed identify a significant relation between TTV viral load and age but did not see a significant effect of gender or CMV serostatus, although the point estimate of the CMV serostatus regression coefficient indicates a similar but non-significant trend. However, due to the differences in study populations and statistical approaches to manage confounding factors, any cross-study comparisons must be done with caution. As to the main hypothesis of this study, there was no association between TTV viral load and AD status and hence no evidence to suggest that TTV DNA, as a biomarker of impaired viral immunity, could serve as a predictor of AD or marker of disease.

Predictors of TTV status was analysed using the assay sensitivity cut-off of 200 copies/ml to separate study subjects into TTV positive and negative populations, without any significant findings. The assumption that DNA negativity indicates that the subject is uninfected could however be flawed if there are TTV infected subjects that are able to control TTV viraemia to a permanent or intermittent non-viraemic state

Regarding host genetic factors related to immune control, HLA-B27 and HLA-B57 have been previously correlated with lower viral load and enhanced disease control in HIV and hepatitis C patients [18, 21]. The selection of these alleles for statistical modelling was based on limitations in the total number of predictor variables allowed, but we do not exclude that other classical and non-classical HLA alleles and genetic variants in the innate immune system such as killer cell immunoglobulin-like receptors (KIRs) could affect TTV immune control. The underlying mechanisms are not completely clear, but it has been previously shown by Elahi et al that CD8-cells restricted by the HLA-B27 and HLA-B57 alleles keep proliferative capacity and evade T_reg_-mediated suppression in chronic viral infections [19, 20]. Our data suggest that the HLA-B27 allele is related to TTV viral load, possibly by enhancing immune control.

In conclusion, our data do not indicate a relationship between AD and TTV immune control but support previous findings suggesting that TTV viral load could be used a marker of impaired viral immunity, possibly relevant for other clinical settings.

## Supporting information

S1 Dataset (XLSX)
